# Supplementary material for: Identification of photoperiod-induced specific miRNAs in the adrenal glands of Sunite sheep (Ovis aries)
Source: Front Vet Sci. 2022 Jul 22;9:888207. doi: 10.3389/fvets.2022.888207 (PMC9354845; doi:10.3389/fvets.2022.888207)
Supplement: Supplementary file 1 [file Data_Sheet_1.ZIP › Supplementary file/miRDeep2 file.zip!/miRDeep2 file/Novel_Structure/SP42A1/Novel_112.pdf]

Provisional ID	:	NC_019482.2_25426
Score total	:	1.7
Score for star read(s)	:	-1.3
Score for read counts	:	0
Score for mfe	:	1.4
Score for randfold	:	1.6
Score for cons. seed	:	
Total read count	:	79
Mature read count	:	79
Loop read count	:	0
Star read count	:	0

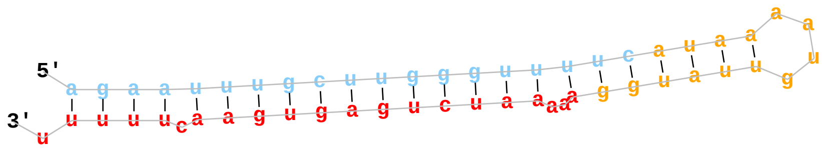

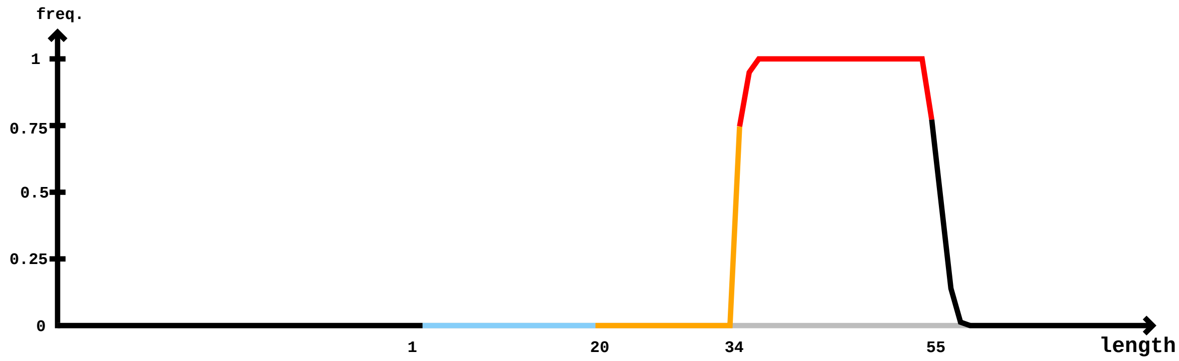

Star Mature

```
5'- aaccauuauauacagaugauuuagugaguuggccaaagaauuugcuuggguuuucauaaaaauguuauggaaaaaucugagugaacuuuuuggccaacggaauauuuuucucc   -3'    exp
    ...(((((.............))))).((((((((((((.((((((((((((((((((((....)))))))..)))))))))))).))))))))))))((((.....))))..   reads   mm   sample
    ......................................................................aaaaaucugaAugaacuuuu.......................   17      1    seq
    ......................................................................aaaaaucugaAugaacuuuuu......................   42      1    seq
    .......................................................................aaaaucugagugaacuuuu.......................   1       0    seq
    .......................................................................aaaaucugagugaacuuuuu......................   4       0    seq
    .......................................................................aaaaCcugagugaacuuuuu......................   1       1    seq
    .......................................................................aaaaucugagugaacuuuuug.....................   1       0    seq
    .......................................................................aaaaCcugagugaacuuuuugg....................   1       1    seq
    .......................................................................aaaaucugagugaacuuuuugg....................   8       0    seq
    ........................................................................aaaucugagugaacuuuuu......................   2       0    seq
    ........................................................................aaaucugagugaacuuuuugg....................   1       0    seq
    ........................................................................aaaCcugagugaacuuuuuggc...................   1       1    seq
```
